# Reply to the letter: “Postintervention pain levels after elective coronary angiography”

**DOI:** 10.1590/1806-9282.20231580

**Published:** 2024-05-03

**Authors:** Raif Kılıç, Tuncay Güzel, Adem Aktan

**Affiliations:** 1 Çermik State Hospital, Department of Cardiology – Diyarbakır, Turkey.; 2 Health Science University, Gazi Yaşargil Training and Research Hospital, Department of Cardiology – Diyarbakır, Turkey.; 3 Mardin Training and Research Hospital, Department of Cardiology – Mardin, Turkey.

Dear editor,

We read with great appreciation the comment made by Engin et al.^1^ for our original article entitled “Comparison of pain levels of traditional radial, distal radial, and transfemoral coronary catheterization^2^”. We appreciate the authors for their interest toward our article and for their time to share their concerns.

In our study, the pain levels were evaluated according to different intervention points in patients who underwent coronary angiography. Our patients were enrolled from 3 centers, and a total of 540 patients were included, with 180 patients in each group. The interventions were performed by the same physician in each center. Angiography was performed through three access points at each center. In all the three centers, femoral access is mostly preferred and distal radial access (DRA) is less commonly used. In approximately 1 year, 180 patients who underwent distal radial access were reached. This number of patients was reached earlier in other patient groups. Since the groups being equal would make the study more valuable, we determined the number of patients to be 180 for each group. During this period, coronary angiography was performed on approximately 6,500 people in 3 centers. The choice of method is left to the patient's preference and the physician's discretion. For example, if the patient is extremely obese, radial access may be preferred, while a weak radial pulse or the presence of an arteriovenous fistula may cause a deviation from radial access.

It is known that the use of analgesics and sedation in patients before the procedure reduces the pain levels^3^. We did not perform any such procedure on our study patients. In the anamnesis we obtained from the patients, we found that they did not routinely use any analgesic or antipsychotic medication. We did not include patients using these medications in the study. The level of anxiety before the procedure is directly proportional to the pain. There are various studies on this subject^4,5^. However, in our study, we focused more on the differences in pain levels at access sites. Therefore, we did not assess the level of anxiety before the procedure.

Doppler ultrasonography (DUSG) can play a pivotal role in improving the success rate of the intervention^6^. Since access to DUSG was not easy enough in the centers where the study was conducted, it was not used routinely in our study. This is stated in the limitations section of the study.

In our study, there were 40 patients in the severe pain group, and 32 of these patients underwent coronary angiography via distal radial artery access. The average number of punctures was approximately two times higher in this patient group than the other groups. However, there was also a higher body mass index and longer processing time and access time in the severe pain group. Therefore, it may not be correct to attribute severe pain only to the number of punctures. To clarify this, we performed univariate and multivariate logistic regression analysis and presented it in Table 1. Accordingly, in multivariate logistic regression analysis, body mass index, processing time, number of punctures and access zone (DRA) were found to be predictors of severe pain.

**Table 1:** Independent determinants of severe pain in patients undergoing coronary angiography in univariate and multivariate logistic regression analysis model.

	Univariate analysis			Multivariate analysis		
	OR	95%CI	p	OR	95%CI	p
Body mass index	0.820	0.752–0.894	**<0.001**	0.814	0.718–0.923	**0.001**
Processing time	1.095	1.068–1.124	**<0.001**	1.097	1.064–1.131	**<0.001**
Access time	1.080	1.056–1.103	**<0.001**	1.022	0.986–1.059	0.237
Number of punctures	5.570	3.415–9.084	**<0.001**	3.232	1.345–7.768	**0.009**
Access zone (DRA)	0.105	0.047–0.234	**<0.001**	0.114	0.040–0.328	**<0.001**

OR: odds ratio; CI: confident interval; DRA: distal radial access.
